# Metagenome-Assembled Genome Sequence of Dolichospermum circinale Strain Clear-D4, Assembled from a Harmful Cyanobacterial Bloom Enrichment Culture

**DOI:** 10.1128/MRA.01123-20

**Published:** 2020-12-03

**Authors:** Kyra M. Florea, J. Cameron Thrash, Eric A. Webb

**Affiliations:** a Department of Biological Sciences, University of Southern California, Los Angeles, California, USA; Georgia Institute of Technology

## Abstract

Dolichospermum circinale (formerly Anabaena circinale) is a significant harmful algal bloom species. We report the draft metagenome-assembled genome (MAG) for a strain of *D. circinale* (Clear-D4) obtained from an enrichment culture. The genome sequence comprises 5,029,933 bp in 560 contigs with a GC content of 37%.

## Abstract (precis)

Dolichospermum circinale (formerly Anabaena circinale) is a significant harmful algal bloom species. We report the draft metagenome-assembled genome (MAG) sequence for a strain of *D. circinale* (Clear-D4) obtained from an enrichment culture. The genome sequence comprises 5,029,933 bp in 560 contigs with 37% GC content.

## ANNOUNCEMENT

Cyanobacterial harmful algal blooms (cyanoHABs) pose environmental, ecological, and economic threats to freshwaters. Species of *Dolichospermum*, a diazotrophic, bloom-forming, heterocystous, cyanobacterial genus, have the potential to produce cyanotoxins, including microcystin, anatoxin, and saxitoxin (1–3). Increasing *Dolichospermum* bloom frequency in recent decades necessitates an improved understanding of this prolific cyanoHAB genus (4). Clear Lake, CA, is 303d-listed for nutrients and experiences annual cyanoHABs (5). To better understand the microbial communities involved in *Dolichospermum* cyanoHABs, we performed metagenomic sequencing on a *Dolichospermum* enrichment culture, resulting in a new metagenome-assembled genome (MAG) for Dolichospermum circinale.

Bucket tow surface water samples were collected from Clear Lake (lat 38.973166, long 122.72809) in August 2019. Free *Dolichospermum* trichomes, visualized with a dissecting scope, were pipettor hand-picked and enriched in 50% BG-11_0_ medium at 25°C with 100 μmol Q/m^2^/s light on a 12:12-h light/dark cycle. Additions of 50% BG-11_0_ were introduced every other week to maintain growth. Prior to sequencing, we identified the genus *Dolichospermum* morphologically in the above enrichments with a Zeiss AxioStar epifluorescence microscope (Oberkochen, Germany) (6). A single enrichment culture (Clear-D4) was chosen for metagenomic sequencing. Cell material from the Clear-D4 enrichment (50 ml) was filtered onto 8-μm polycarbonate filters and rinsed into 2-ml bead-beating tubes using lysing solution from the Qiagen DNeasy PowerBiofilm kit (Hilden, Germany). The cells were lysed using 5 liquid N_2_ freeze-thaw cycles, followed by the addition of proteinase K and incubation at 55°C overnight. Genomic DNA was then extracted using the aforementioned Qiagen kit protocol. The quality of isolated DNA was verified using Tris-borate-EDTA (TBE) gel electrophoresis and quantified via NanoDrop UV-visible (UV-Vis) spectroscopy and Qubit spectrofluorimetry (Thermo Fisher Scientific, Waltham, MA). Library preparation (i.e., NEBNext DNA library prep kit via the manufacturer’s recommendations) and 1 Gbp of Illumina paired-end (PE) 2 × 150-bp sequencing were conducted by Novogene (Nanjing, China) with 300-bp size-selected inserts, generating 19,844,532 reads. KBase and modules therein were used for *de novo* assembly with default settings unless otherwise noted (7). Prior to *de novo* assembly, the quality of the paired-end reads was checked with FastQC v0.11.5 (8), and the reads were trimmed to enhance quality using Trimmomatic v0.36 (9). *De novo* genome assembly was done using metaSPAdes v3.13.0 (10). Binning was completed using MaxBin2 v2.2.4 (11), with refinement using anvi-refine in Anvi’o (12). Genome annotation was completed using PGAP with default settings (13). Initial bin taxonomy was determined using GTDB-tk in the PhyloSanity wrapper (14).

Clear-D4 consisted of 560 contigs, 5,029,933 bp, a GC content of 37%, and an *N*_50_ value of 52,616 bp. A total of 4,813 coding genes were identified (12). CheckM v1.0.18 (15) (with default settings) estimated this initial MAG to be 91.85% complete with 6.47% contamination. The refined genome was phylogenetically confirmed as *Dolichospermum circinale* using GTDB-tk v1.1.1 db_r95 using “classify_wf.” The predicted metabolism of Clear-D4 was analyzed using the FuncSanity module of MetaSanity (14) with default settings, which projected that this organism is a diazotrophic, oxygenic photoautotroph that can reduce arsenate and produce sulfolipids. Screening for secondary metabolites with antiSMASH revealed the predicted presence of geosmins; however, no cyanobacterial toxins were detected by this analysis (16).

### Data availability.

This whole-genome shotgun project has been deposited at DDBJ/ENA/GenBank under the accession number JACVZX000000000. The version described in this paper is the first version, JACVZX010000000. The BioProject number is PRJNA657201, and the reads are available at the SRA under accession number SRX8961729.

## References

[1] Sivonen K, Namikoshi M, Evans WR, Carmichael WW, Sun F, Rouhiainen L, Luukkainen R, Rinehart KL 1992 Isolation and characterization of a variety of microcystins from seven strains of the cyanobacterial genus Anabaena. Appl Environ Microbiol 58:2495–2500. doi:10.1128/AEM.58.8.2495-2500.1992. 1514796 PMC195810

[2] Rantala-Ylinen A, Känä S, Wang H, Rouhiainen L, Wahlsten M, Rizzi E, Berg K, Gugger M, Sivonen K 2011 Anatoxin—a synthetase gene cluster of the cyanobacterium Anabaena sp. strain 37 and molecular methods to detect potential producers. Appl Environ Microbiol 77:7271–7278. doi:10.1128/AEM.06022-11. 21873484 PMC3194866

[3] Pereyra JPA, D'Agostino PM, Mazmouz R, Woodhouse JN, Pickford R, Jameson I, Neilan BA 2017 Molecular and morphological survey of saxitoxin-producing cyanobacterium Dolichospermum circinale (Anabaena circinalis) isolated from geographically distinct regions of Australia. Toxicon 138:68–77. doi:10.1016/j.toxicon.2017.08.006. 28797629

[4] Li X, Dreher TW, Li R 2016 An overview of diversity, occurrence, genetics and toxin production of bloom-forming Dolichospermum (Anabaena) species. Harmful Algae 54:54–68. doi:10.1016/j.hal.2015.10.015. 28073482

[5] State Water Resources Control Board. 2011 Final 2014/2016 California Integrated Report [Clean Water Act Section 303(d) List/305(b) Report].

[6] Komárek J, Zapomìlová E 2008 Planktic morphospecies of the cyanobacterial genus Anabaena = subg. Dolichospermum—2. Part: straight types. Fottea 8:1–14. doi:10.5507/fot.2008.001.

[7] Arkin AP, Cottingham RW, Henry CS, Harris NL, Stevens RL, Maslov S, Dehal P, Ware D, Perez F, Canon S, Sneddon MW, Henderson ML, Riehl WJ, Murphy-Olson D, Chan SY, Kamimura RT, Kumari S, Drake MM, Brettin TS, Glass EM, Chivian D, Gunter D, Weston DJ, Allen BH, Baumohl J, Best AA, Bowen B, Brenner SE, Bun CC, Chandonia J-M, Chia J-M, Colasanti R, Conrad N, Davis JJ, Davison BH, DeJongh M, Devoid S, Dietrich E, Dubchak I, Edirisinghe JN, Fang G, Faria JP, Frybarger PM, Gerlach W, Gerstein M, Greiner A, Gurtowski J, Haun HL, He F, Jain R, Joachimiak MP, Keegan KP, Kondo S, Kumar V, Land ML, Meyer F, Mills M, Novichkov PS, Oh T, Olsen GJ, Olson R, Parrello B, Pasternak S, Pearson E, Poon SS, Price GA, Ramakrishnan S, Ranjan P, Ronald PC, Schatz MC, Seaver SMD, Shukla M, Sutormin RA, Syed MH, Thomason J, Tintle NL, Wang D, Xia F, Yoo H, Yoo S, Yu D 2018 KBase: the United States Department of Energy Systems Biology Knowledgebase. Nat Biotechnol 36:566–569. doi:10.1038/nbt.4163. 29979655 PMC6870991

[8] Andrews S 2010 FastQC: a quality control tool for high throughput sequence data. Babraham Bioinformatics, Cambridge, United Kingdom.

[9] Bolger AM, Lohse M, Usadel B 2014 Trimmomatic: a flexible trimmer for Illumina sequence data. Bioinformatics 30:2114–2120. doi:10.1093/bioinformatics/btu170. 24695404 PMC4103590

[10] Nurk S, Meleshko D, Korobeynikov A, Pevzner PA 2017 MetaSPAdes: a new versatile metagenomic assembler. Genome Res 27:824–834. doi:10.1101/gr.213959.116. 28298430 PMC5411777

[11] Wu Y-W, Simmons BA, Singer SW 2016 MaxBin 2.0: an automated binning algorithm to recover genomes from multiple metagenomic datasets. Bioinformatics 32:605–607. doi:10.1093/bioinformatics/btv638. 26515820

[12] Eren AM, Esen OC, Quince C, Vineis JH, Morrison HG, Sogin ML, Delmont TO 2015 Anvi’o: an advanced analysis and visualization platform for ’omics data. PeerJ 3:e1319. doi:10.7717/peerj.1319. 26500826 PMC4614810

[13] Tatusova T, DiCuccio M, Badretdin A, Chetvernin V, Nawrocki EP, Zaslavsky L, Lomsadze A, Pruitt KD, Borodovsky M, Ostell J 2016 NCBI Prokaryotic Genome Annotation Pipeline. Nucleic Acids Res 44:6614–6624. doi:10.1093/nar/gkw569. 27342282 PMC5001611

[14] Neely CJ, Graham ED, Tully BJ 2020 MetaSanity: an integrated microbial genome evaluation and annotation pipeline. Bioinformatics 36:4341–4344. doi:10.1093/bioinformatics/btaa512. 32426808 PMC7520038

[15] Parks DH, Imelfort M, Skennerton CT, Hugenholtz P, Tyson GW 2015 CheckM: assessing the quality of microbial genomes recovered from isolates, single cells, and metagenomes. Genome Res 25:1043–1055. doi:10.1101/gr.186072.114. 25977477 PMC4484387

[16] Blin K, Shaw S, Steinke K, Villebro R, Ziemert N, Lee SY, Medema MH, Weber T 2019 antiSMASH 5.0: updates to the secondary metabolite genome mining pipeline. Nucleic Acids Res 47:W81–W87. doi:10.1093/nar/gkz310. 31032519 PMC6602434

